# Wide-range tuning of interfacial exchange coupling between ferromagnetic Au/Co and ferrimagnetic Tb/Fe(Co) multilayers

**DOI:** 10.1038/s41598-018-35042-x

**Published:** 2018-11-15

**Authors:** Łukasz Frąckowiak, Piotr Kuświk, Maciej Urbaniak, Gabriel David Chaves-O’Flynn, Feliks Stobiecki

**Affiliations:** 1 Institute of Molecular Physics, Polish Academy of Sciences, ul. Smoluchowskiego 17, 60-179 Poznan, Poland; 2 Westchester Community College, State University of New York, 75 Grasslands Road, Valhalla, New York 10595 USA

## Abstract

The ability to perform wide-range tuning of the magnetic field required to switch the magnetization of ferromagnetic layers with perpendicular magnetic anisotropy is of great importance for many applications. We show that, for (Au/Co)_2(3)_ multilayers, this field can be changed from minus several kOe to plus several kOe because of changes to the coupling with a ferrimagnetic multilayer [either (Tb/Fe)_6_ or (Tb/Co)_6_] across a Au spacer (either homogeneous 1 nm thick or wedge-shaped). The adjustable parameters are the ratio of sublayer thicknesses of the ferrimagnet and the sequence of layers around the Au spacer. The change of the sequence from Co/Au/Co to Tb/Au/Co is accompanied by both the reduction of the interaction energy and the change of the magnetic field sign necessary to switch the magnetization of ferromagnetic multilayers. For a 1 nm thick Au spacer this fields change from positive (negative) to negative (positive) if the ferrimagnet is dominated by the transition metal (rare earth) as a result of its composition. The characteristic oscillatory behavior of RKKY-like coupling is demonstrated using a system with a wedge-shaped Au spacer.

## Introduction

Great interest in amorphous ferrimagnetic (FI) rare rarth (RE) - transition metal (TM) alloy films was caused initially by their potential application in bubble memories^1^ and later, in magneto-optical memories^2–4^. Particularly important for these applications are the strong perpendicular magnetic anisotropy (PMA) and the possibility to easily manipulate magnetization, anisotropy, compensation temperature, and Curie temperature by changing the composition of the alloy. In the 1980’s, it was shown that RE/TM multilayers (MLs) with sublayers of small thickness exhibit properties similar to RE-TM alloy films^5–15^. Specifically, Tb based structures, despite having low Curie temperature (*T*_C_ = 220 K) also exhibit ferrimagnetic properties at room temperature (RT). Note that the RE-TM alloys with Tb as the RE element are sperimagnets^16–20^, however, in the literature they are usually referred as ferrimagnets due to the antiferromagnetic coupling between the TM and the RE sublayers^8,9,12,14,21^. The properties of these synthetic ferrimagnets differ strongly from RE-TM alloys if the sublayers are thick^6,10^, however, for thin sublayers the differences between the alloy films and MLs are negligible^10,22^. This occurs for thicknesses *t*_Fe_ ≤ 0.75 nm, *t*_Tb_ ≤ 1.1 nm for Fe/Tb; and *t*_Co_ = *t*_Tb_ ≤ 1.5 nm for Co/Tb. For both systems the amorphous structure is preserved and similar dependence of magnetic properties on average composition is observed.

Nowadays, RE-TM alloy films are intensively investigated because of new promising applications. For example, they have been used in all-optical switching (AOS)^23–25^, and they have been used to achieve spin-orbit torque (SOT) in systems composed of a FI layer coupled with a metal layer^26–28^. Furthermore, RE-TM films inside a soft/hard ferrimagnetic structure^29–33^ or a ferromagnetic/ferrimagnetic (F/FI) bilayer^16,34,35^, can be used to modify the magnetic field required to switch magnetization direction of F or/and FI layers or to facilitate AOS^36^. Similar to interactions observed in ferrimagnetic materials, in the F/FI system the interactions between the TM spins of F and FI layers favors a parallel configuration; and the interaction between the RE spins of the FI layer and TM spins of the F layer, an antiparallel configuration. This interaction, in contrast to the exchange interaction between the antiferromagnetic/ferromagnetic (AF/F) layers (commonly used in spintronics devices), shows important advantages. One of them is the possibility to achieve strong coupling in layered systems with PMA. Further advantages are related to the insensitivity of the F/FI system to structural defects, such as grain boundaries or interface roughness^37^. Therefore, in F/FI systems a strong PMA can be obtained together with an exchange interaction that is tunable over a wide range of strengths^38^. The interlayer coupling across the nonmagnetic spacer was also studied independently of the investigations of the direct coupling between F and FI layers in F/FI systems^36,39–41^. It was demonstrated that, depending on the spacer’s material, these coupling can be relatively strong for Cu^40,41^ and Ru^39^ or weak for Ta^40^. Moreover, the characteristic RKKY-like coupling oscillations with varying Ru thickness were experimentally observed^39^. The results concerning interlayer coupling in simple trilayer systems have been inconclusive. For example, studies on Fe/Au/Tb MLs^42^ described a slowly decaying oscillatory coupling for Au thicknesses smaller than 2 nm. In contrast, experiments on Co/Cu/Gd and Co/Y/Gd MLs^43^ indicate a strong monotonic decay of interlayer coupling as spacer thickness increases.

In this work, we report on the magnetic properties of F/Au/FI layered systems where F is (Au/Co)_3_ and FI is either (Tb/Co)_6_ or (Tb/Fe)_6_ MLs. Thicknesses of the sublayers were adjusted to ensure PMA in F and FI multilayers. We determined the influence of the Tb sublayers thickness (average nominal concentration of Tb) on the magnetization reversal of the whole system and on the interaction between the FI and the F structures. We show that the choice of layers which are in direct contact with the Au spacer has a profound influence on the interaction between the F and the FI layers. Changing the materials lying next to the 1 nm thick Au spacer from TM/Au/TM to RE/Au/TM leads not only to a strong decrease in the exchange coupling strength but also to a change of its sign (transition from ferromagnetic to antiferromagnetic coupling).

The paper is organized as follows. First, we describe the properties of FI MLs made of Tb wedges interspersed with either Co or Fe of constant thickness. Second, we describe how each of these two types of FI ML interact with (Au/Co) F ML. For these studies we consider two different scenarios: in one case the (Au/Co) and the (Tb/Fe(Co)) are in direct contact; in the second case, they are separated by an additional Au layer. We will point out that the insertion of this additional Au layer changes the way the multilayers couple together during magnetization reversal. The specific Au layer that plays the role of spacer is different in both scenarios and can be distinguished by the materials adjacent to it: Co/Au/Co in one case or Co/Au/Tb in other. In the final part of the paper we describe the transition between these two scenarios based on measurements of wedge-shaped Au spacers.

## Results and Discussion

### Ferrimagnetic Tb/Co and Tb/Fe multilayers

**Figure 1:**
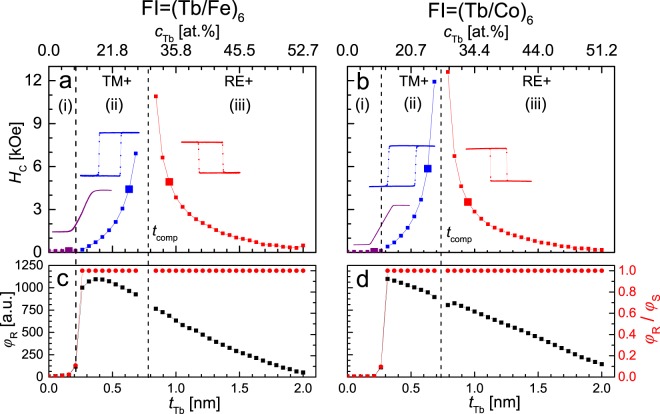
Coercitivity (*H*_C_) (**a**,**b**), Kerr signal at remanence (*φ*_R_) and ratio *φ*_R_/*φ*_S_ (*φ*_S_ − Kerr rotation at saturation) (**c**,**d**) versus thickness of Tb sublayers (*t*_Tb_) and concentration (*c*_Tb_) for (Tb-*t*_Tb_/Fe-0.66 nm)_6_ (**a**,**c**) and (Tb-*t*_Tb_/Co-0.66 nm)_6_ (**b**,**d**) multilayers. Large points in (**a**) and (**b**) correspond to inserted hysteresis loops.

The studies of the exchange interaction in F/Au/FI layered systems were preceded by precise characterization of magnetic properties of the (Tb-wedge 0–2 nm/Fe-0.66 nm)_6_ and (Tb-wedge 0–2 nm/Co-0.66 nm)_6_ MLs. The ratios of Tb (0 ≤ *t*_Tb_ ≤ 2 nm) and Co or Fe (*t*_Co_ = *t*_Fe_ = 0.66 nm) sublayers thicknesses were chosen to cover the concentration range corresponding to the transition from easy plane anisotropy (EPA) to PMA and to the compensation point (concentration at which the magnetic moments of the RE and TM sublayers are compensated). To guarantee that the magnetic properties of RE/TM MLs were similar to those of RE-TM alloy films, we chose relatively small thicknesses of the sublayers^6,10^. Figure 1 shows the coercive field (*H*_C_), the ratio of Kerr rotation in remanence in relation to its saturation value (*φ*_R_/*φ*_S_) and *φ*_R_ as a function of *t*_Tb_. In all these curves, three characteristic ranges of *t*_Tb_ can be distinguished. They are characterized by: (i) EPA − (*φ*_R_)/(*φ*_S_) ≈ 0, (ii) PMA − (*φ*_R_)/(*φ*_S_) = 1 and predominance of the TM sublayers, which indicates that the magnetization of the TM sublayers in saturation is parallel to the magnetic field (hereinafter this will be denoted as TM+), (iii) PMA and predominance of the RE (RE+) sublayers. Ranges (ii) and (iii) are separated at *t*_Tb_ corresponding to the compensation point (*t*_Tb_ = *t*_comp_). At *t*_Tb_ = *t*_comp_ the resultant saturation magnetization (*M*_S_) equals zero (*M*_S_ = 0)^37^ and *H*_C_(*t*_Tb_) shows a singularity. In other words, as *t*_Tb_ approaches *t*_comp_ from both thinner (TM+ range) and thicker (RE+ range) Tb sublayers, *H*_C_ increases to infinity. This is in accordance with the equation *H*_C_ ≈ *K*_eff_/*M*_S_ (Eq. (1) from M. Tang *et al*.^38^). Therefore, *H*_C_ data are not available near *t*_comp_ due to the limits of available magnetic field *H*_max_ (±15 kOe).

The average Tb concentrations (*c*_Tb_) were determined for specific *t*_Tb_, and corresponding TM layer thicknesses (*t*_Fe_ or *t*_Co_), using the formula given in F. Richomme *et al*.^21^. The compensation is at *t*_comp_ = 0.79 nm (*c*_Tb_ = *c*_comp_ = 30.5 at.%) and *t*_comp_ = 0.74 nm (*c*_comp_ = 27.8 at.%) for the structure (Fe/Tb)_6_ and (Co/Tb)_6_, respectively. These values are higher than for the alloy films^38,44^. This is a typical effect for RE/TM MLs, because for thicker Tb sublayers some part of the layer may be in a paramagnetic state^10^. The *φ*_R_(*t*_Tb_) for *t*_Tb_ corresponding to PMA shows a monotonic decrease. The approximately linear dependence indicates that the P-MOKE signal is mainly associated with the TM sublayers^24^. The signal reduction with increasing *t*_Tb_ results from light absorption in the wedge-shaped Tb sublayers. Since the TM elements dominate the Kerr signal, the hysteresis loops are inverted at the compensation point (Fig. 1)^45^. The results presented in Fig. 1 demonstrate thus the essential similarities in magnetic properties between RE/TM MLs and RE-TM alloy films.

### Magnetization reversal and interlayer coupling in F/Au/FI layered systems

In the previous part of the paper we have shown that, due to a strong AF coupling between the Fe(Co) and Tb sublayers, the magnetization reversal of the whole (Tb/Fe(Co))_6_ FI MLs takes place simultaneously. In ferromagnetic (Au-1 nm/Co-0.8 nm)_N_ MLs for *N* = 2 or 3 the hysteresis loop is rectangular (i.e. the ratio of remanence to saturation magnetizations equals one, *M*_R_/*M*_S_ = 1) and the magnetization reversal also occurs simultaneously for all Co sublayers. However, in the latter case it is because of an effective ferromagnetic coupling between the Co sublayers^46,47^.

**Figure 2:**
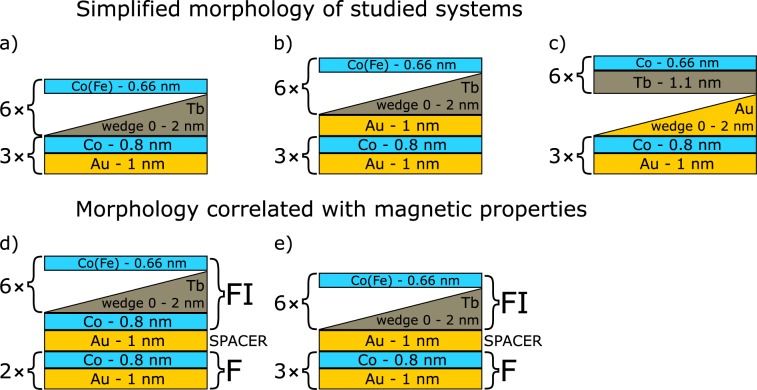
Morphology of investigated systems consisting of (Au-1 nm/Co-0.8 nm)_3_ and (Tb-wedge 0–2 nm/Fe(Co)-0.66 nm)_6_ multilayers in direct contact (**a**), separated by Au-1 nm spacer (**b**), in (**c**) (Au-1 nm/Co-0.8 nm)_3_ is separated from (Tb-1.1 nm/Co-0.66 nm)_6_ with a wedge-shaped Au-0–2 nm spacer. In panels (**d**) and (**e**), the structures of systems (**a**) and (**b**) are described differently to emphasize their magnetization reversal behavior. Note that in (**d**) and (**e**) the Au spacer possesses different surroundings. The system presented in panel (c) exhibits the transition between the cases presented in panels (**d**) and (**e**) for *t*_Au_ = 0.25 nm (see text).

**Figure 3:**
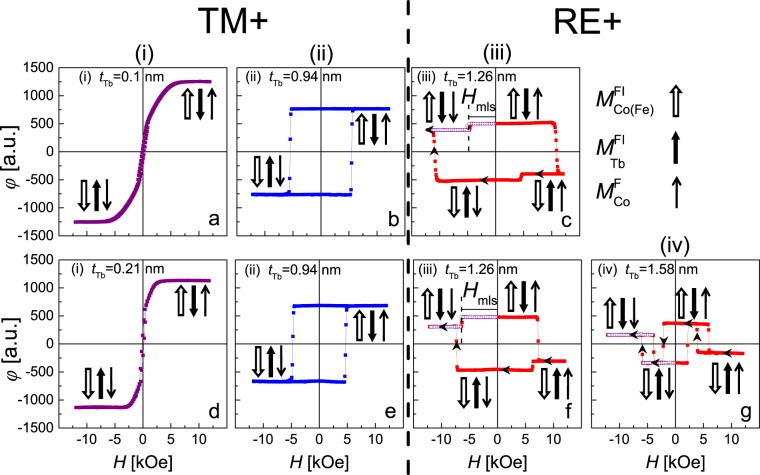
Representative, for particular ranges of Tb sublayer thicknesses (see Fig. 4), P-MOKE hysteresis loops for structures F/Au-1 nm/FI with F = (Au-1 nm/Co-0.8 nm)_2_ and FI = Co-0.8 nm/(Tb-*t*_Tb_/Fe-0.66 nm)_6_ (**a**–**c**) and FI = Co-0.8 nm/(Tb-*t*_Tb_/Co-0.66 nm)_6_ (**d**–**g**). The Tb thicknesses given for each panel correspond to the large points in dependencies presented in Fig. 4(a,b).

We have investigated three groups of F/Au/FI systems with structures schematically presented in Fig. 2. The difference between the first two structures is that the system presented in Fig. 2b contains an additional Au layer as compared to the system in Fig. 2a. In the simplest form the morphology of both types of samples can be described as follows: F/FI (Fig. 2a) and F/Au-1 nm/FI (Fig. 2b) where F = (Au-1 nm/Co-0.8 nm)_3_ and FI = (Tb-wedge/Co(Fe)-0.66 nm)_6_. However, from a magnetization reversal point of view this description is misleading. Neglecting the *t*_Tb_ range corresponding to FI MLs with EPA (Fig. 3a,d), the magnetization reversal of the F and the FI MLs forming the system presented in Fig. 2a,d takes place simultaneously for *t*_Tb_ < *t*_comp_ (Fig. 3b,e), however, for *t*_Tb_ > *t*_comp_ the reversal is sequential (Fig. 3c,f). Analysis of the P-MOKE signal in the *t*_Tb_ range corresponding to sequential reversal of F and FI MLs indicates that only two Co-0.8 nm sublayers take part in the reversal of the F layer. Thus, the reversal of the Co-0.8 nm sublayer adjacent to the Tb wedge takes place together with the FI MLs (Fig. 2d). This shows that one Co-0.8 nm layer is more strongly coupled with the sublayers forming the FI ML than with the remaining two Co-0.8 nm layers belonging to the F ML. Therefore, a more appropriate description of the system in Fig. 2a is: F/Au-1 nm/FI where F = (Au-1 nm/Co-0.8 nm)_2_, and FI = Co-0.8 nm/(Tb-wedge 0–2 nm/Fe-0.66 nm)_6_ or FI = Co-0.8 nm/(Tb-wedge 0–2 nm/Co-0.66 nm)_6_ (Fig. 2d).

If we consider that an additional Co-0.8 nm sublayer is now part of FI ML, the *c*_Tb_ value as a function of *t*_Tb_ needs to be recalculated. As a consequence of this, the compensation point expressed in Tb thickness shift rightwards. Now *t*_comp_ = 1.13 nm (*c*_comp_ = 34.1 at.%) and *t*_comp_ = 1.08 nm (*c*_comp_ = 32.0 at.%) for systems with FI = Co/(Tb/Fe)_6_ and FI = Co/(Tb/Co)_6_, respectively. The *c*_Tb_ values given in parenthesis here already take into account the additional Co sublayer of the FI MLs. Note that, despite this correction, the Tb concentrations corresponding to the compensation point are still higher (about 4 at.%) than for isolated FI layer (Fig. 1). This is probably caused by the interaction between the FI and the F structures (in further discussion it will be demonstrated that this interaction is ferromagnetic). A similar effect is described by M. Tang *et al*.^38^.

**Figure 4:**
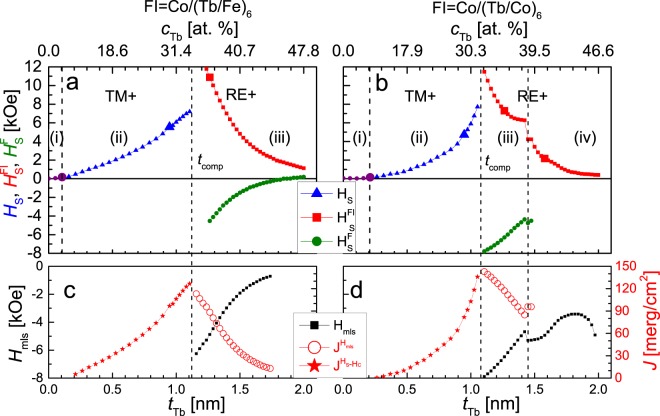
Switching fields (*H*_S_) of entire system (triangles), ferromagnetic (${H}_{{\rm{S}}}^{{\rm{F}}}$) (circles) and ferrimagnetic (${H}_{{\rm{S}}}^{{\rm{FI}}}$) (squares) multilayers (**a**,**b**), large points in panels (a) and (b) correspond to hysteresis loops presented in Fig. 3, minor loop shift (*H*_mls_) and interlayer exchange energy (*J*) (**c**,**d**) as a function of Tb sublayer thickness (*t*_Tb_) and concentration (*c*_Tb_) for F/Au-1 nm/FI systems with F = (Au-1 nm/Co-0.8 nm)_2_. The left side (**a**,**c**) shows results for FI = Co-0.8 nm/(Tb-*t*_Tb_/Fe-0.66 nm)_6_ and the right side (**b**,**d**), for FI = Co-0.8 nm/(Tb-*t*_Tb_/Co-0.66 nm)_6_ multilayers. The interlayer coupling energy is calculated using equation (1) with *H*_C_ = 250 Oe (stars) and equation (2) (open circles).

The analysis of the magnetization reversal process, (*φ*(*H*) presented in Fig. 3) allows to distinguish three characteristic *t*_Tb_ ranges marked in Fig. 4a for the (Au-1 nm/Co-0.8 nm)_3_/(Tb-wedge/Fe-0.66 nm)_6_ system and four ranges for the (Au-1 nm/Co-0.8 nm)_3_/(Tb-wedge/Co-0.66 nm)_6_ (Fig. 4b) system. In both cases, the first three ranges can be characterized in the same way. In range (i) (Fig. 3a,d) the shape of the hysteresis loop is typical of layered systems consisting of weakly coupled films with EPA and PMA, which in our case corresponds to FI and F MLs, respectively^35,48,49^. For larger *t*_Tb_ (in ranges (ii–iv)) both the F and FI MLs exhibit PMA. The FI MLs are TM+ in range (ii) (Fig. 3b,e), and RE+ in ranges (iii) and (iv) (Fig. 3c,f,g). In range (ii), due to the strong ferromagnetic interaction between F and FI MLs, the reversal of both MLs occurs simultaneously. We identify the field necessary to reverse the “isolated” magnetic ML in the interacting F/Au/FI system as the switching field *H*_S_ instead of *H*_C_. Thus, as mentioned before, for range (ii) ${H}_{S}={H}_{{\rm{S}}}^{{\rm{F}}}={H}_{{\rm{S}}}^{{\rm{FI}}}$ (*H*_S_, ${H}_{{\rm{S}}}^{{\rm{F}}}$, ${H}_{{\rm{S}}}^{{\rm{FI}}}$ denote switching fields of the entire system, F MLs, and FI MLs, respectively). Because of the ferromagnetic interaction between F and FI, ${H}_{{\rm{C}}}^{{\rm{F}}}\le {H}_{S}\le {H}_{{\rm{C}}}^{{\rm{FI}}}$ where ${H}_{{\rm{C}}}^{{\rm{F}}}$ and ${H}_{{\rm{C}}}^{{\rm{FI}}}$ denote *H*_C_ of single F and FI MLs, respectively. The increase of *H*_S_ with *t*_Tb_ results from increasing strength of ferromagnetic coupling, as *t*_Tb_ approaches *t*_comp_, due to the compensation of the FI sublattices^33^. In the *t*_Tb_ range corresponding to the simultaneous magnetization reversal, the interlayer coupling energy can be determined from equation proposed by V. Grolier *et al*.^50^

1 $$J=({H}_{{\rm{S}}}-{H}_{{\rm{C}}}^{{\rm{F}}}){M}_{{\rm{S}}}^{{\rm{Co}}}{t}_{{\rm{F}}}$$

(note that in this case the *H*_C_ value must be determined from a separate measurement). In range (iii) and (iv), i.e., for *t*_Tb_ corresponding to the FI ML RE+, at a magnetic field large enough to saturate the entire F/A/FI system (*H*_sat_ ≤ |*H*_max_|) the magnetization of the RE (TM) sublattice is parallel (antiparallel) to *H* (Fig. 3c,f). This configuration is energetically unfavorable because of the ferromagnetic interaction between the Co sublayers located on each side of the Au spacer. Therefore, in the *t*_Tb_ range corresponding to strong ferromagnetic coupling the magnetization reversal of the F ML takes place at a magnetic field oriented in the same direction as *H*_max_ applied at the start of the hysteresis loop [at ${H}_{{\rm{S}}}^{{\rm{F}}}$ > 0 (${H}_{{\rm{S}}}^{{\rm{F}}}$ < 0) for positive (negative) *H*_max_]. With increasing *t*_Tb_ (decreasing *J*) ${H}_{{\rm{S}}}^{{\rm{F}}}$ crosses zero and then approaches ${H}_{{\rm{C}}}^{{\rm{F}}}$. In the field range between the reversal of the F ML and the reversal of the FI ML (|${H}_{{\rm{S}}}^{{\rm{F}}}$| ≤ |*H*| ≤ |${H}_{{\rm{S}}}^{{\rm{FI}}}$|) the magnetic moment of Co sublayers in direct contact with the Au spacer are parallel to each other, what is energetically favorable for F and FI MLs coupled ferromagnetically (Fig. 3c,f). As a consequence, for the F/Au/FI system with FI RE+ the magnitude of switching and coercive fields obeys the relation |${H}_{{\rm{S}}}^{{\rm{FI}}}$| ≥ ${H}_{{\rm{C}}}^{{\rm{FI}}}$. This is also evident from the distinct asymmetry of *H*_S_(*t*_Tb_) with respect to *t*_Tb_ = *t*_comp_ (Fig. 4a,b).

For the case of sequential magnetization reversal the sign and the value of *J* can be determined from the minor loop shifts (*H*_mls_ parameter) (Fig. 3c,f). The following expression relates *J* and *H*_mls_ in F/spacer/F or F/spacer/FI systems:

2 $$J=-\,{\rm{sgn}}[{H}_{{\rm{\max }}}\times ({t}_{{\rm{comp}}}-{t}_{{\rm{Tb}}})]\times {H}_{{\rm{mls}}}{M}_{{\rm{S}}}t,$$

where *M*_S_ and *t* refer to the free layer. The sign (−) in the right side of the equation is explicitly written to highlight the opposite relation between *H*_max_ and *H*_mls_^51^. The (*t*_comp_ − *t*_Tb_) factor is included so that expression becomes valid for all values of *t*_Tb_^16,39^. The dependencies of *H*_mls_ and *J* on *t*_Tb_ (Fig. 4c,d) confirm the previous conclusion about enhancement of interlayer coupling in the vicinity of the compensation concentration of FI MLs.

For the F/Au/FI system with FI = Co/(Tb/Co)_6_ and *t*_Tb_ ≥ 1.45 nm (range iv) the FI ML is the free layer and F is the pinning layer. The representative hysteresis loop is shown in Fig. 3g. It can be assumed that the structure and magnetic properties of the F ML do not change with *t*_Tb_. Therefore, the change of the magnetization reversal sequence of F and FI layers between range (iii) and (iv) observed for the F/Au/FI system with FI = Co(Tb/Co)_6_ and the lack of such a change for the system with FI = Co(Tb/Fe)_6_, are probably associated with different magnetic properties of the Tb/Co and Tb/Fe MLs. In Fig. 2b in ref.^3^, changes of *M*_S_(x) for Tb_1−x_Co_x_ and Tb_1−x_Fe_x_ alloys are presented. These measurements show clearly different behavior for both systems in *c*_Tb_ range above the compensation point (for *c*_Tb_ > *c*_comp_). For both systems, as *c*_Tb_ increases (x decreases) *M*_S_ initially increases and then starts to decrease. *M*_S_ becomes zero at concentration *c*_crit_ corresponding to the transition from ferrimagnetic to paramagnetic properties. However, the *c*_Tb_ range between *c*_comp_ and *c*_crit_ (*c*_comp_ ≤ *c*_Tb_ ≤ *c*_crit_) is much larger for Tb-Fe (over 30 at.%) than for Tb-Co (about 15 at.%). For small thicknesses of sublayers, the magnetic properties of RE/TM multilayers and alloy films are similar. Therefore, most probably the transition from range (iii) to (iv) observed in Fig. 4b at *t*_Tb_ ≈ 1.45 nm is related to the strong *M*_S_ reduction for higher *t*_Tb_. Note that due to the strong ferromagnetic coupling between F and FI layers, at the second stage of the reversal process (after magnetization reversal of FI), a simultaneous magnetization reversal of the F and FI MLs takes place (Fig. 3g).

To summarize the results described above, we emphasize that, for the F/Au/FI system with F = (Au-1 nm/Co-0.8 nm)_2_, *H*_S_ can be tuned in the range from minus several kOe to plus several kOe by an appropriate selection of *t*_Tb_. This effect was achieved by coupling the F layer with the FI layer across a 1 nm thick Au spacer surrounded by Co-0.8 nm sublayers (Fig. 2d). Note that the increase of interlayer coupling due to an insertion of a Co layer between the Cu spacer and the FI layer in GdCo/Cu/Co structures was recently shown by A.V. Svalov and coworkers^41^.

**Figure 5:**
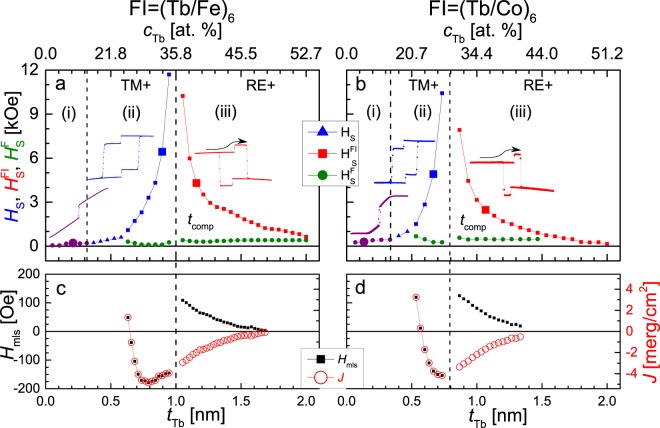
Switching fields (*H*_S_) of the entire system (triangles), ferromagnetic (circles) and ferrimagnetic (squares) (**a**,**b**), large symbols in panels (a,b) correspond to inserted hysteresis loops, minor loop shift (*H*_mls_) and interlayer exchange energy (*J*) (**c**,**d**) as a function of Tb sublayer thickness (*t*_Tb_) and concentration (*c*_Tb_) for: F/Au-1 nm/FI systems with F = (Au-1 nm/Co-0.8 nm)_3_ and FI = (Tb-*t*_Tb_/Fe-0.66 nm)_6_ (**a**,**c**), FI = (Tb-*t*_Tb_/Co-0.66 nm)_6_ (**b**,**d**) multilayers.

**Figure 6:**
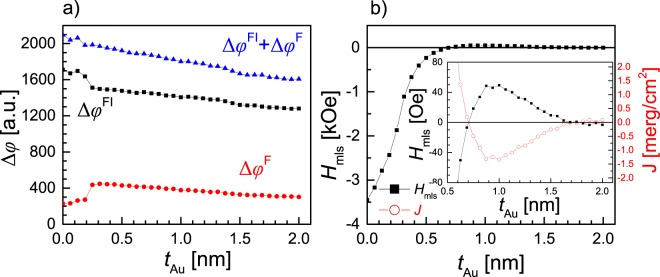
Results of P-MOKE measurements performed for (Au-1 nm/Co-0.8 nm)_3_/Au-wedge 0-2 nm/(Tb-1.1 nm/Co-0.66 nm)_6_ layered system. (**a**) Kerr signal related to reversal of: ferromagnetic multilayer (Δ*φ*^F^), ferrimagnetic multilayer (Δ*φ*^FI^), and (Δ*φ*^F^ + Δ*φ*^FI^) as a function of Au spacer thickness (*t*_Au_). (**b**) Minor loop (related to reversal of ferromagnetic multilayer) shift and energy of interlayer exchange coupling vs. *t*_Au_. Note that one of Co-0.8 nm sublayers reverses together with ferrimagnetic or ferromagnetic multilayer for *t*_Au_ < 0.25 nm and *t*_Au_ > 0.25 nm, respectively.

To gather more information on coupling in F/Au/FI systems, the structure presented in Fig. 2a was modified by inserting an additional 1 nm thick Au sublayer (Fig. 2b,e). The changes of the P-MOKE signal indicate that the magnetization reversal of all three Co-0.8 nm layers constituting the ferromagnetic part of the system takes place simultaneously. The careful analysis of P-MOKE signal related to reversal of F ML for loops presented in Fig. 3c,f and hysteresis loops shown in Fig. 5 corresponds to reversal of two and three Co-0.8 nm sublayers, respectively (see also Fig. 6a). The dependencies of *H*_S_, ${H}_{{\rm{S}}}^{{\rm{F}}}$, ${H}_{{\rm{S}}}^{{\rm{FI}}}$(*t*_Tb_) and *H*_mls_(*t*_Tb_) for (Au/Co)_3_/Au/(Tb/Fe)_6_ and (Au/Co)_3_/Au/(Tb/Co)_6_ systems are shown in Fig. 5. A comparison of the data from Figs 4 and 5 shows that the Au layer insertion results in an extension of the *t*_Tb_ range in which the sequential reversal of the F and FI MLs takes place. For both systems for which the measurement results are presented in Fig. 5, in the *t*_Tb_ range corresponding to the sequential reversal of F and FI MLs, the free layer is the F = (Au/Co)_3_ ML. The ${H}_{{\rm{S}}}^{{\rm{F}}}$(*t*_Tb_) dependence shows only slight changes (within limits not exceeding ±200 Oe), indicating a weak coupling between F and FI MLs. The *H*_mls_(*t*_Tb_) and *J*(*t*_Tb_) dependencies obtained from minor loops presented in Fig. 5c,d confirm that the coupling between F and FI MLs in the structure shown in Fig. 2b,e is much weaker (about thirty times) than that seen in Fig. 2a,d. Additionally, the type of interaction (sign of *J*) is now antiferromagnetic (*J* < 0). However, in the TM+ range, with decreasing *t*_Tb_ the strengths of antiferromagnetic coupling decreases, and for *t*_Tb_ = 0.65 nm for FI = (Tb/Fe) and *t*_Tb_ = 0.55 nm for FI = (Tb/Co) a change to ferromagnetic coupling occurs. These behaviors suggest that for small Tb thickness the Co spins belonging to F MLs interact both with Fe(Co) and Tb spins of the FI MLs. In the RE+ range (*t*_Tb_ > *t*_comp_) *J* decreases monotonically with *t*_Tb_. The maximum value of antiferromagnetic interaction occurs for *t*_Tb_ = 0.8 nm; that is, below *t*_comp_. This shift is probably caused by the antiferromagnetic interaction between F and FI MLs.

Considering the small difference in morphology of systems presented in Fig. 2a,d and in Fig. 2b,e such strong changes of coupling strengths and sign may be surprising. Therefore, for a more detailed explanation of the difference in the magnetic properties of the systems shown in Fig. 2, an F/Au-wedge/FI layered system, similar to those presented in Fig. 2b but with wedge-shaped Au spacer instead, was deposited and characterized with P-MOKE measurements. The investigation was restricted to the system in which the (Tb-1.1 nm/Co-0.66 nm)_6_ MLs constituted its FI part. The thickness of Tb sublayers, *t*_Tb_ = 1.1 nm, was chosen to ensure that FI MLs were of the RE+ type and in consequence sequential magnetization reversal of F and FI MLs takes place. This was crucial for the analysis of the interlayer coupling between both of the MLs. Figure 6a shows the *t*_Au_ dependence of the P-MOKE signal related to the reversal of the entire system (Δ*φ*) and separately for the F (Δ*φ*^F^) and the FI (Δ*φ*^FI^) constituent MLs. This result shows that at *t*_Au_ ≈ 0.25 nm a transition occurs from a situation in which the Co-0.8 nm sublayer located closest to the FI ML undergoes magnetization reversal together with FI (for *t*_Au_ ≤ 0.25 nm) to a situation in which its reversal takes place together with the other two Co-0.8 nm sublayers forming F ML. In other words, the wedge-shaped Au sublayer is the spacer if its thickness (*t*_Au_) is greater than 0.25 nm, otherwise the spacer is the upper Au sublayer sandwiched between two Co-0.8 nm sublayers. The *H*_mls_(*t*_Au_) dependence shown in Fig. 6b is non-monotonic. For *t*_Au_ ≤ 0.7 nm, *H*_mls_ ≤ 0 Oe, which indicates ferromagnetic coupling with a strength that increases with decreasing *t*_Au_. The *H*_mls_ reaches maximum value, corresponding to the antiferromagnetic coupling, for *t*_Au_ ≈ 1 nm and then decreases, going to negative values (ferromagnetic coupling) for *t*_Au_ ≥ 1.9 nm. The *J*(*t*_Au_), dependence is similar to the one observed when a non-magnetic Au spacer separates the ferromagnetic layers^46,50^. However, according to results reported for Co/Cu/Gd and Co/Y/Gd^43^ structures and considering that, in the discussed system, the Au spacer for *t*_Au_ ≥ 0.25 nm is also between TM and RE sublayers (in our case between Co and Tb) we should expect an antiferromagnetic coupling with monotonically decreasing strength with spacer thickness. On the other hand, E. Shypil^42^ observed an oscillatory coupling with slight decrease of coupling amplitude in a Co/Au/Tb system. Our results presented in Fig. 6b strongly suggest that, despite surrounding the Au spacer with Co and Tb, the interlayer coupling is mainly mediated by the Co sublayers, where one of these sublayers belongs to the F ML and the second one to the FI ML. This interpretation is also supported by the fact that, in the vicinity of *t*_Au_ ≈ 0.25 nm, the *J*(*t*_Au_) dependence does not show any anomalies. Despite the fact that the coupling is mainly mediated by Co sublayers, the existence of the Tb sublayer (between Au spacer and Co-0.66 nm sublayer belonging to the FI MLs) may have distinct influence on the coupling between F and FI MLs. In particular the coupling strengths can be reduced and the *J*(*t*_Tb_) dependence can be modified when a part of Tb sublayers is paramagnetic^10^. Among all the different effects described above, that should be considered in the interpretation of Fig. 6b, we believe that the RKKY-like interaction plays a significant role in the coupling between F and FI MLs across the Au spacer.

### Summary

We report on investigations of systems composed of ferromagnetic F = Au/Co and ferrimagnetic FI = Tb/Fe(Co) multilayers exhibiting perpendicular magnetic anisotropy. We studied the magnetization reversal process and the coupling between F and FI across a 1 nm thick Au spacer as a function of the Tb sublayers thickness. We showed that, because of coupling between the F and FI layers, the switching fields of the F multilayer can be tuned in a wide range (from minus several kOe to plus several kOe) by changing the thickness of Tb sublayers (average Tb concentration) in the FI multilayer. We also showed that to obtain a strong exchange coupling it is required to have a Au spacer separating the F and FI structures placed between the ferromagnetic (Co in our case) sublayers. For such a structure, with a Au spacer 1 nm thick, the interlayer coupling is ferromagnetic with maximal strengths at Tb thickness corresponding to the compensation point at which the switching field of F MLs changes sign. However, for the Au-1 nm spacers placed between Tb and Co sublayers the coupling is antiferromagnetic and much weaker. The oscillatory behavior of coupling was observed for ferrimagnetic and ferromagnetic multilayers interacting across wedge-shaped Au spacer suggesting existence of the RKKY-like coupling.

## Experimental Section

### Samples deposition

The layered systems were deposited from elemental targets using magnetron sputtering in an ultra-high vacuum chamber (base pressure 10^−9^ mbar) with an argon pressure of 10^−3^ mbar on naturally oxidized Si(100) substrates coated with a Ti-4 nm/Au-29 nm buffer layer, which previously proved suitable as a buffer for Au/Co MLs^46^. The growth of films was carried out at RT in the field of a permanent magnet (3 kOe) oriented perpendicular to the substrate.

### Magnetic measurements

Magnetic properties were investigated *ex situ* at RT using a magneto-optical Kerr effect in polar configuration (P-MOKE). The magnetic field (*H*) was applied perpendicularly to the sample’s plane with a maximum value of *H*_max_ = ±15 kOe. For P-MOKE investigations a laser with 655 nm wavelength and spot size of 0.2 mm was used. The changes in magnetic properties as a function of the Tb thickness (wedge-shaped sublayers) were investigated by moving the sample relative to a stationary light beam.

## Data Availability

The data of this study are available from the corresponding authors on reasonable request.

## References

[1] Kobliska R, Gangulee A, Cox D, Bajorek C (1977). Temperature dependence of the magnetic properties of amorphous Co-Gd-Mo thin films. IEEE Transactions on Magn..

[2] Kryder MH (1985). Magneto-optic recording technology (invited). J. Appl. Phys..

[3] Hansen P, Clausen C, Much G, Rosenkranz M, Witter K (1989). Magnetic and magneto-optical properties of rare-earth transition-metal alloys containing Gd, Tb, Fe, Co. J. Appl. Phys..

[4] Cheng S-N, Kryder M, Mathur M (1989). Stress related anisotropy studies in DC-magnetron sputtered TbCo and TbFe films. IEEE Transactions on Magn..

[5] Freitag AE, Chowdhury AR (1999). Magnetic properties of Fe/Tb multilayers with large Fe layer thickness. J. Appl. Phys..

[6] Richomme F (1996). Magnetic anisotropy in amorphous Fe/Tb multilayers. J. Magn. Magn. Mater..

[7] Richomme, F., Teillet, J., Fnidiki, A. & Keune,W. Structural and magnetic properties of UHV-evaporated Fe/Tb multilayers: Effect of the substrate temperature. *Phys. Rev. B* **64**, 10.1103/PhysRevB.64.094415 (2001).

[8] Sato N (1986). Magnetic properties of amorphous Tb-Fe thin films with an artificially layered structure. J. Appl. Phys..

[9] Shan ZS, Sellmyer DJ (1990). Magnetism of rare-earth–transition-metal nanoscale multilayers. I. Experiments on Dy/Co, Dy/Fe, and Tb/Fe. Phys. Rev. B.

[10] Ertl L, Endl G, Hoffmann H (1992). Structure and magnetic properties of sputtered Tb/Co multilayers. J. Magn. Magn. Mater..

[11] Yang F, He T, Chen JB, Pan F (2002). Transition of ferromagnetism to superparamagnetism in Fe/Tb multilayers. J. Appl. Phys..

[12] Šmakov J, Lapinskas S, Tornau E, Rosengren A (1998). Magnetization and compensation temperature of transitionmetal–rare-earth multilayers in a model with long-range interactions. J. Magn. Magn. Mater..

[13] Garreau G, Farle M, Beaurepaire E, Kappler J (1998). Spin-reorientation phase transition in Co/Tb and Co/Ho ultrathin films. J. Magn. Magn. Mater..

[14] Svalov AV, Savin PA, Kurlyandskaya GV, Gutiérrez J, Vas’kovskiy VO (2002). Spin-valve magnetoresistive structures based on Co/Tb multilayer films. Tech. Phys..

[15] Mangin S (2014). Engineered materials for all-optical helicity-dependent magnetic switching. Nat. Mater..

[16] Schubert, C. *et al*. Interfacial exchange coupling in Fe-Tb/[Co/Pt] heterostructures. *Phys. Rev. B* 87, 10.1103/PhysRevB.87.054415 (2013).

[17] Vas’kovskiy VO, Svalov AV, Balymov KG, Kulesh NA (2012). Effect of annealing on the magnetic anisotropy and hysteretic properties of film structures containing Tb-Co amorphous layers. The Phys. Met. Metallogr..

[18] Soltani M, Chakri N, Lahoubi M (2001). Composition and annealing dependence of magnetic properties in amorphous Tb–Co based alloys. J. Alloy. Compd..

[19] Kim W-S, Andrä W, Kleemann W (1998). Influence of interfaces on the perpendicular magnetic anisotropy in Tb/Fe multilayers. Phys. Rev. B.

[20] Mangin, S. *et al*. Magnetization reversal in exchange-coupled GdFe/TbFe studied by x-ray magnetic circular dichroism. *Phys. Rev. B* **70**, 10.1103/PhysRevB.70.014401 (2004).

[21] Richomme F, Teillet J, Fnidiki A, Auric P, Houdy P (1996). Experimental study of the structural and magnetic properties of Fe/Tb multilayers. Phys. Rev. B.

[22] Sato N, Habu K (1987). Amorphous rare-earth–transition-metal thin films with an artificially layered structure. J. Appl. Phys..

[23] Alebrand S (2012). Light-induced magnetization reversal of high-anisotropy TbCo alloy films. Appl. Phys. Lett..

[24] Hassdenteufel A (2014). Dependence of all-optical magnetic switching on the sublattice magnetization orientation in Tb-Fe thin films. Appl. Phys. Lett..

[25] El Hadri MS, Hehn M, Malinowski G, Mangin S (2017). Materials and devices for all-optical helicity-dependent switching. J. Phys. D: Appl. Phys..

[26] Finley, J. & Liu, L. Spin-Orbit-Torque Efficiency in Compensated Ferrimagnetic Cobalt-Terbium Alloys. *Phys. Rev. Appl*. **6**, 10.1103/PhysRevApplied.6.054001 (2016).

[27] Ueda K, Mann M, Pai C-F, Tan A-J, Beach GSD (2016). Spin-orbit torques in Ta/TbxCo_100−x_ ferrimagnetic alloy films with bulk perpendicular magnetic anisotropy. Appl. Phys. Lett..

[28] Je S-G (2018). Spin-orbit torque-induced switching in ferrimagnetic alloys: Experiments and modeling. Appl. Phys. Lett..

[29] Becker S, Luciński T, Rohrmann H, Stobiecki F, Röll K (1995). Exchange coupled double layer films (ECDLs) consisting of Tb/Fe multilayer stacks. J. Magn. Magn. Mater..

[30] Mangin, S., Montaigne, F. & Schuhl, A. Interface domain wall and exchange bias phenomena in ferrimagnetic/ferromagnetic bilayers. *Phys. Rev. B* **68**, 10.1103/PhysRevB.68.140404 (2003).

[31] Lin C-C, Lai C-H, Jiang R-F, Shieh H-PD (2003). High interfacial exchange energy in TbFeCo exchange-bias films. J. Appl. Phys..

[32] Henry, Y., Mangin, S., Hauet, T. & Montaigne, F. Positive exchange-bias induced by interface domain wall quenching in Gd Fe/Tb Fe films. *Phys. Rev. B* **73**, 10.1103/PhysRevB.73.134420 (2006).

[33] Radu, F., Abrudan, R., Radu, I., Schmitz, D. & Zabel, H. Perpendicular exchange bias in ferrimagnetic spin valves. *Nat. Commun*. **3**, 10.1038/ncomms1728 (2012). 10.1038/ncomms1728 22395606

[34] Hebler, B. *et al*. Influence of the Fe-Co ratio on the exchange coupling in TbFeCo/[Co/Pt] heterostructures. *Phys. Rev. B* **93**, 10.1103/PhysRevB.93.184423 (2016).

[35] Romer S (2012). Temperature dependence of large exchange-bias in TbFe-Co/Pt. Appl. Phys. Lett..

[36] Gorchon J (2017). Single shot ultrafast all optical magnetization switching of ferromagnetic Co/Pt multilayers. Appl. Phys. Lett..

[37] Schubert Christian (2014). Magnetic Order and Coupling Phenomena.

[38] Tang, M. H. *et al*. Interfacial exchange coupling and magnetization reversal in perpendicular [Co/Ni]N/TbCo composite structures. *Sci. Reports* **5**, 10.1038/srep10863 (2015). 10.1038/srep10863 PMC4466588 26074295

[39] Lin M-S, Lai C-H (2007). Perpendicular interlayer coupling through oscillatory Ruderman-Kittel-Kasuya-Yosida interaction between Co/Pt multilayers and Co/TbCo bilayers. J. Appl. Phys..

[40] Tang M, Zhang Z, Jin Q (2015). Manipulation of perpendicular exchange bias effect in [Co/Ni] N/(Cu, Ta)/TbCo multilayer structures. AIP Adv..

[41] Svalov A, Vas’kovskiy V, Orue I, Kurlyandskaya G (2017). Tailoring of switching field in GdCo-based spin valves by inserting Co layer. J. Magn. Magn. Mater..

[42] Shypil E, Pogorily A, Podyalovsky D (2004). Oscillations and change of sign in indirect exchange coupling of Fe/Au/Tb trilayer structures. Low Temp. Phys..

[43] Takanashi K, Fujimori H, Kurokawa H (1993). Indirect exchange coupling through nonmagnetic metal spacers in Co/X/Gd multilayers (X = Cu and Y). J. Magn. Magn. Mater..

[44] Hassdenteufel A (2013). Thermally Assisted All-Optical Helicity Dependent Magnetic Switching in Amorphous Fe_100−x_Tb_x_ Alloy Films. Adv. Mater..

[45] Alebrand, S. *et al*. Subpicosecond magnetization dynamics in TbCo alloys. *Phys. Rev. B* **89**, 10.1103/PhysRevB.89.144404 (2014).

[46] Matczak M (2013). Antiferromagnetic magnetostatic coupling in Co/Au/Co films with perpendicular anisotropy. J. Appl. Phys..

[47] Urbaniak M, Stobiecki F, Gaul A, Ehresmann A (2015). Magnetization reversal of Co/Au multilayer stripes with keV-He^+^ ion bombardment induced coercivity gradient. J. Phys. D: Appl. Phys..

[48] Urbaniak, M. *et al*. Domain-Wall Movement Control in Co/Au Multilayers by He+-Ion-Bombardment-Induced Lateral Coercivity Gradients. *Phys. Rev. Lett*. **105**, 10.1103/PhysRevLett.105.067202 (2010). 10.1103/PhysRevLett.105.067202 20868002

[49] Gong WJ (2011). Tuning exchange bias in ferromagnetic/ferromagnetic/antiferromagnetic heterostructures [Pt/Co]/NiFe/NiO with in-plane and out-of-plane easy axes. J. Appl. Phys..

[50] Grolier V (1993). Unambiguous evidence of oscillatory magnetic coupling between Co layers in ultrahigh vacuum grown Co/Au(111)/Co trilayers. Phys. Rev. Lett..

[51] Baltz V (2009). Balancing interlayer dipolar interactions in multilevel patterned media with out-of-plane magnetic anisotropy. Appl. Phys. Lett..

